# Validation of the Italian Version of the Visual Function and Corneal Health Status (V-FUCHS) Questionnaire: A Patient-Reported Visual Disability Instrument for Fuchs’ Endothelial Corneal Dystrophy

**DOI:** 10.3390/jcm14113996

**Published:** 2025-06-05

**Authors:** Matilde Buzzi, Alberto Carnicci, Martina Maccari, Silvia Magherini, Sanjay V. Patel, Gianni Virgili, Fabrizio Giansanti, Rita Mencucci

**Affiliations:** 1 Eye Clinic, Department of Neuroscience, Psychology, Pharmacology and Child Health (NEUROFARBA), University of Florence, Largo Brambilla 3, 50134 Florence, Italy; maccarimartina3@gmail.com (M.M.); magherini.silvia@gmail.com (S.M.); gianni.virgili@unifi.it (G.V.); fabrizio.giansanti@unifi.it (F.G.); rita.mencucci@unifi.it (R.M.); 2 Unit of Ophthalmology, Department of Medicine, Surgery and Neuroscience, University of Siena, Viale Mario Bracci 16, 53100 Siena, Italy; dr.albertocarnicci@gmail.com; 3 Department of Ophthalmology, Mayo Clinic, 200 First Street SW, Rochester, MN 55905, USA; patel.sanjay@mayo.edu

**Keywords:** Fuchs’ endothelial corneal dystrophy, glare, questionnaire, patient-reported outcomes, endothelial keratoplasty

## Abstract

**Background/Objectives**: The Visual Function and Corneal Health Status (V-FUCHS) questionnaire assesses vision-related quality of life in patients affected by Fuchs endothelial corneal dystrophy (FECD) through 15 items, which are divided into the “visual acuity (VA) Factor” and “glare Factor” domains. The purpose of this study was to translate and validate the Italian version of the V-FUCHS instrument. **Methods**: The original V-FUCHS questionnaire was translated into Italian using certified forward and backward translation methods and administered to patients with FECD undergoing a unilateral or bilateral Descemet membrane endothelial keratoplasty (DMEK) for FECD and healthy controls. Its test–retest reliability was assessed by administering the questionnaire twice, four weeks apart. Modified Krachmer grade, best-corrected visual acuity (BCVA) and central corneal thickness (CCT) were also measured. **Results**: A total of 74 participants, aged 45 to 83 years, were prospectively enrolled and divided into five subgroups: mild-to-moderate FECD (*n* = 18), advanced FECD (*n* = 15), unilateral DMEK (*n* = 9), bilateral DMEK (*n* = 12), and healthy controls (*n* = 20). Retest reliability confirmed the consistency and agreement of their responses (intraclass correlation coefficient > 0.90 for both factors). The Italian V-FUCHS effectively discriminated between different stages of FECD severity, with significant differences in VA and glare factor scores across all subgroups (*p* < 0.001). While both BCVA and CCT showed correlations with V-FUCHS scores, only the association between the VA factor and BCVA was statistically significant (*p* < 0.05), indicating that the VA factor meaningfully reflects patients’ measured VA. **Conclusions**: The proposed Italian version of the V-FUCHS questionnaire is a valid and reliable tool for assessing visual disability in patients with FECD before and after DMEK. This instrument may aid in optimizing endothelial keratoplasty timings and evaluating postoperative symptomatic improvements in FECD patients.

## 1. Introduction

Fuchs’ endothelial corneal dystrophy (FECD) is the most prevalent form of primary corneal endothelial dystrophy and constitutes the leading indication for corneal transplantation globally [1,2]. The disease is characterized by a gradual loss of corneal endothelial cells (CECs) and the development of focal excrescences, known as guttae, on Descemet’s membrane. These alterations cause progressive endothelial dysfunction, leading to corneal edema and gradually declining vision [1]. With the advancement of endothelial keratoplasty techniques, such as Descemet membrane endothelial keratoplasty (DMEK), that enable earlier treatment in patients with FECD, the accurate identification of early signs and symptoms has become crucial for optimal surgical timing [3]. Conventional anatomical and optical parameters, such as CEC density, central corneal thickness (CCT), slit-lamp biomicroscopy grading, and high-contrast visual acuity, are often not adequate to stage the disease reliably, particularly in its early stages [4,5]. Structural corneal changes precede the clinical symptoms of corneal edema and are associated with increased intra-corneal light scatter, which typically presents as disability glare, one of the earliest subjective complaints reported by patients [6,7]. As a result, the relevance of patient-reported outcome measures (PROMs) that can capture the symptomatic burden of the disease and its impairment of daily activities has increased [8]. PROMs play a critical role in patient-centered care models and personalized medicine by integrating the patient’s perspective into clinical decision-making, helping tailor interventions to individual needs and optimizing treatment timing and outcomes [9]. Several questionnaires have been developed to assess vision-related quality of life and visual impairment [10,11,12,13]. These patient-reported outcome measures have not, however, been specifically designed for FECD and therefore lack the construct validity necessary to accurately reflect disease-specific visual impairments, such as glare sensitivity and diurnal fluctuations in visual function [14,15]. The Visual Function and Corneal Health Status (V-FUCHS) questionnaire, originally created by Wacker et al., is a 15-item, FECD-specific instrument designed to quantify self-reported visual disability. The questionnaire contains 7 items on visual acuity (VA) (VA factor) and 8 items on glare sensitivity and diurnal variation in visual function (glare factor) [16]. It uniquely addresses not only visual difficulties, but also glare and diurnal fluctuations in vision, which are often among the earliest and most troubling symptoms reported by patients with FECD.

Validated translations of the V-FUCHS are already available in German [17], Spanish [18], and French [19], facilitating its integration into clinical practice across diverse linguistic and cultural settings. To our knowledge, no Italian-language questionnaire specifically designed to assess visual symptoms in patients with FECD exists. The objective of this study was to develop an Italian version of the V-FUCHS questionnaire and to validate its use among Italian-speaking patients with FECD before and after DMEK.

## 2. Materials and Methods

### 2.1. Study Population

This prospective study was conducted in accordance with the Declaration of Helsinki and received approval from the Hospital Ethics Committee (Comitato Etico Regionale per la Sperimentazione Clinica della Toscana—sezione AREA VASTA CENTRO, protocol number 25170_OSS, date of approval 14 November 2023). Verbal and written informed consent were obtained from all participants prior to enrollment, after a clear explanation of the nature of the study. Native Italian-speaking patients were prospectively recruited from the Careggi University Hospital (Florence, Italy) Cornea Service between December 2023 and December 2024. The inclusion criterion was a clinically documented diagnosis of FECD, with or without prior DMEK. In patients without prior DMEK, severity of their FECD was graded using slit-lamp biomicroscopy with a modified Krachmer scale [20,21], based on central and paracentral guttae distribution, guttae density, and the presence of clinically evident corneal edema. Patients were stratified into five groups: (1) mild-to-moderate FECD (modified Krachmer scale 1 to 4) (*n* = 18), (2) advanced FECD (modified Krachmer scale 5 and 6) (*n* = 15), (3) unilateral DMEK (*n* = 9), (4) bilateral DMEK (*n* = 12), and (5) healthy controls (*n* = 20). The inclusion criteria for healthy controls were an age within the range of 45 to 85 years, an absence of clinical signs of FECD, and no endothelial alterations on corneal endothelial microscopy performed with Perseus (Costruzione Strumenti Oftalmici, Florence, Italy). Included patients were either pseudophakic or phakic with a LOCS III (Lens Opacities Classification System III) grading of ≤2 [22]. Pseudophakic patients either exhibited no visually significant posterior capsule opacification or had previously undergone YAG laser capsulotomy. Patients with non-FECD ocular conditions affecting the endothelium, a history of intraocular surgery other than phacoemulsification, or conditions affecting the quality of their vision (e.g., macular degeneration, glaucoma) were excluded. Individuals with cognitive impairment or an inability to comprehend the questionnaire were also excluded.

### 2.2. Translation of the V-FUCHS Questionnaire

The V-FUCHS translation was performed according to internationally approved standards [23]. The Italian version of the V-FUCHS questionnaire was developed in cooperation with a professional coordinator, two certified forward translators, and one certified backward translator. All of them were qualified professional medical translators with training and qualification in ophthalmic terminology to ensure semantic accuracy and the clinical relevance of the translated content. The two forward translators independently translated the questionnaire from English to Italian; concordant items were accepted directly, while discrepancies were resolved through team discussion. The completed Italian version was then back-translated into English by a masked certified translator, and any inconsistencies were solved through the same consensus-based process. A notable case of discrepancy occurred in the translation of the term “starburst”, used to describe a visual phenomenon experienced by patients. While one translator proposed a literal rendering, the final consensus translation was “circondate di raggi luminosi” (surrounded by bright rays), which was considered more accessible to patients and more properly reflective of the symptom’s appearance. Special care was taken to employ clear, patient-centered language in the Italian V-FUCHS questionnaire to make it understandable and congruent with the patient’s perspective.

### 2.3. Physical Examination and Questionnaire Administration

In the clinical evaluation, best-corrected visual acuity (BCVA) was assessed using the logarithm of the minimum angle of resolution (logMAR) scale, providing a standardized measure of visual function. At the same time, CCT was quantified by optical coherence tomography (OCT)-based anterior segment tomography with MS-39 (Costruzione Strumenti Oftalmici, Florence, Italy). Patients were provided with standardized instructions that specifically accounted for their visual impairment when completing the questionnaire. Each item was rated on a five-point Likert scale ranging from 0 (“Never” or “No difficulty”) to 4 (“All of the time” or “Extreme difficulty”), with higher scores indicating a greater frequency or severity of the perceived limitation.

### 2.4. Reliability and Validity Assessment

The calculations of the V-FUCHS factors and the assessment of their validity and reliability were conducted following the methodology outlined for the development of the V-FUCHS questionnaire [16]. The evaluation was performed using Rasch model-based mean values of the VA and glare factors from the V-FUCHS on a logit scale. Each unidimensional factor was analyzed independently. Item response theory methods, including Rasch model-based analyses, enable the identification and quantification of the underlying latent traits of participants [24,25]. In this approach, items of varying difficulty are assigned different weights, facilitating a more accurate assessment of participants with varying levels of limitations. This provides a clear advantage over classical test theory, which assumes all items to be of the same difficulty [10]. To check whether the data conformed to the expectations of the Rasch model, the goodness-of-fit statistic was assessed using both infit and outfit mean square (MNSQ) statistics. Values between 0.7 and 1.3 are generally considered acceptable [24]. Mean item difficulty and corresponding standard errors were calculated. The calibration of the V-FUCHS questionnaire was further examined using a person–item map to identify items whose difficulty levels were misaligned, either too low or too high, in comparison to the distribution of the latent traits within the FECD population. Test–retest reliability was assessed by administering the questionnaire twice, with the second time approximately 4 weeks after the initial assessment (range: 3.7–4.4 weeks). The second administration was conducted either by mail (*n* = 29) or by telephone call (*n* = 45), depending on participant availability and preference. All 74 participants completed both tests. Reliability was evaluated in terms of absolute agreement and consistency through the intraclass correlation coefficient (ICC). The internal consistency of the questionnaire was estimated using Cronbach’s alpha for the two factors, where values above 0.9 were considered indicative of excellent consistency [26]. To confirm that each item contributed significantly to its own factor, a sub-analysis was conducted by sequentially removing one item at a time. Raw scores for both dimensions were calculated by summing the Likert-scale ratings (0 to 4) on relevant items. Given the good correspondence with Pearson correlations between raw and latent scores (r > 0.9 with a *p*-value of <0.05), and the greater practicality of raw scores in clinical settings, subsequent analyses were based on raw scores. The predictive validity of the questionnaire was also tested by comparing mean VA and glare factor scores among the five subgroups. A one-way analysis of variance (ANOVA) with subsequent pairwise *t*-tests was performed to determine whether the Italian version of the V-FUCHS questionnaire could reliably distinguish between different stages of disease severity. To evaluate the correlation between the questionnaire and clinical measurements (BCVA and CCT), analyses were based on the patient’s better-seeing eye, as determined by VA, given its greater influence on overall visual perception and glare sensitivity. Univariate correlation analysis was conducted between the BCVA and CCT measurements, producing correlation coefficients (0–1) and corresponding *p*-values, where values closer to 1 indicate a stronger association. All statistical analyses were conducted using Jamovi software, version 2.6.26 [27]. Item response theory models were implemented using the R packages eRM [28].

## 3. Results

A total of 74 participants completed the Italian version of V-FUCHS questionnaire, including 33 patients with varying stages of FECD; 21 patients who had undergone DMEK for FECD, either unilaterally or bilaterally; and 20 healthy controls. The cohort included 38 females and 36 males, with a mean age of 66.7 ± 9.4 years. The demographic characteristics, key clinical parameters, and group distribution of the patient population enrolled are listed in Table 1. The visual acuity factor and glare factor’s mean item difficulties and respective standard errors are listed in Table 2. Both VA factor and glare factor were distributed normally. Infit and outfit statistics were within the expected range of 0.5 to 1.5 defined by the Rasch model, confirming the construct validity of the questionnaire [29].

**Figure 1:**
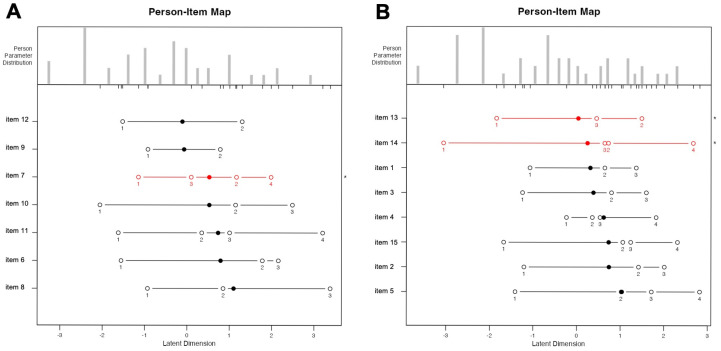
Person–item maps for the visual acuity (**A**) and glare (**B**) factors of the Italian version of the V-FUCHS questionnaire. Scores on the *x*-axis represent the underlying latent trait, ranging from low to high levels of FECD-related disability. The person parameter distribution (upper row) illustrates the number of patients across the spectrum of disability levels. For each item, mean scores are represented by black circles, while hollow circles indicate the threshold scores marking transitions between adjacent response categories. * indicates items in which threshold inversion between categories 2 and 3 occurred.

Our person–item map (Figure 1) indicated that the instrument was generally well-calibrated to the ability distribution of the FECD patient population. However, patients with no-to-very low visual disability, placed on the left side of the histogram, were represented by fewer items compared to those with moderate-to-high levels of disability. For the VA factor, a threshold inversion between categories 2 and 3 was observed in item 7; for the glare factor, a similar inversion occurred for items 13 and 14. In items exhibiting a threshold inversion between categories 2 and 3, patients were more likely to shift from “Rarely” or “A little” to “Most of the time” or “A lot” instead of progressing gradually from “Rarely” or “A little” to “Sometimes” or “Moderate”. Test–retest evaluation confirmed adequate agreement and consistency, with an ICC of 0.88 (95% CI, 0.81–0.92) for the VA factor and 0.91 (95% CI, 0.85–0.93) for the glare factor [30]. To establish internal consistency, Cronbach’s alpha coefficients were estimated, demonstrating excellent reliability both for the VA factor (*α* = 0.90) and for the glare factor (*α* = 0.92). Item deletion analysis did not provide any substantial increase in Cronbach’s alpha, indicating that all items contributed positively to the measurement of both dimensions.

**Figure 2:**
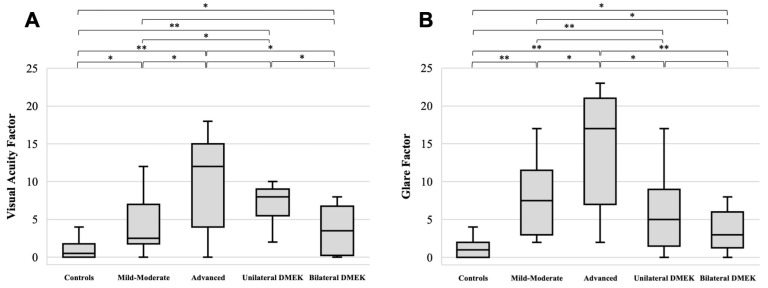
Box-and-whisker plots illustrating the content domains of the V-FUCHS instrument. The distribution of raw scores for the visual acuity (**A**) and glare (**B**) factors is presented across different FECD severity groups, highlighting variations in patient-reported outcomes according to their disease stage and illustrating pairwise comparisons and their respective *p*-values (* *p* < 0.05 and ** *p* < 0.001). The 5 groups were mild-to-moderate FECD (*n* = 18), advanced FECD (*n* = 15), unilateral DMEK (*n* = 9), bilateral DMEK (*n* = 12), and healthy controls (*n* = 20).

To evaluate the predictive validity of the questionnaire, mean scores for the VA and glare factors were compared across the five clinically defined subgroups, reflecting increasing levels of disease severity (Figure 2). A one-way analysis of variance (ANOVA) revealed statistically significant variation among subgroups for both the VA and glare factors (*p* < 0.001 for both), indicating that patient-reported outcomes varied consistently with the progression of disease and following either unilateral or bilateral DMEK. Post hoc pairwise *t*-tests further confirmed that the Italian version of the V-FUCHS questionnaire was able to discriminate between sequential stages of the disease, with significantly higher scores observed in more severe FECD patients. Indeed, most pairwise comparisons revealed statistically significant differences (*p* < 0.05), confirming the discriminative ability of the questionnaire among clinical subgroups, with only four exceptions. Regarding VA scores, no statistically significant differences were observed between patients with advanced FECD and those with unilateral DMEK, as well as between patients with mild-to-moderate FECD and those with bilateral DMEK. Similarly, for glare factor scores, no statistically significant differences were found between patients who had undergone unilateral DMEK and those with either bilateral DMEK or mild-to-moderate FECD (Figure 2). Univariate analyses revealed weak-to-moderate correlations between the questionnaire scores and clinical parameters. The VA factor was correlated with BCVA (*r* = 0.244) and CCT (*r* = 0.124), and the glare factor also demonstrated correlations with BCVA (*r* = 0.217) and CCT (*r* = 0.168). However, only the correlation between the VA factor and BCVA reached statistical significance (*p* < 0.05).

## 4. Discussion

This study aimed to validate the first Italian-language translation of the V-FUCHS instrument as a reliable tool for assessment of patient-reported visual disability in patients with FECD. Consistent with the original English version and previous validations in French, Spanish, and German [16,17,18,19], our findings confirm the preservation of the questionnaire’s two-factor structure, i.e., its VA and glare domains, and support its construct and content validity within an Italian-speaking population. Its adequate test–retest reliability indicated that patients were able to provide consistent and repeatable responses to the questionnaire. In terms of internal consistency, our results showed excellent reliability both for the VA and glare factors, with all items positively contributing to the measurement of both dimensions. The predictive validity of the questionnaire was supported by mean test scores that corresponded to the increasing levels of disease severity across the subgroups, as defined by their modified Krachmer grade [20,21]. Patient-reported outcomes consistently reflected disease progression and changes following either unilateral or bilateral DMEK, showing that the Italian V-FUCHS can successfully discriminate between different stages of the disease.

Person–item maps (Figure 1) indicated that the instrument was generally well calibrated to the ability distribution of the target patient population. An interesting observation emerged in the response patterns for specific questionnaire items, where a threshold inversion was noted between categories 2 and 3 (item 7 for the VA factor; items 13–14 for the glare factor). Specifically, patients were more likely to report a shift from “Rarely” directly to “Most of the time”, bypassing “Sometimes”, as well as from “A little” to “A lot”, bypassing “Moderate”. This non-linear response trend may reflect the heightened sensitivity of patients who are newly experiencing visual disturbances. At the early onset of symptoms, individuals may perceive the impact as disproportionately burdensome, leading to abrupt changes in self-reported severity rather than gradual transitions, or to an overestimation of visual symptoms because of a fear of not being selected for surgery. Such patterns suggest that PROMs like V-FUCHS can detect early functional impairments that may be underestimated or missed by objective clinical tests. This ability to capture subjective visual difficulties, such as glare sensitivity and diurnal fluctuation, even in the absence of measurable changes in BCVA or CCT, underscores the tool’s potential utility for identifying early-stage FECD and supporting timely clinical interventions.

Importantly, our cohort included patients who underwent both unilateral and bilateral DMEK surgery, allowing us to evaluate the responsiveness of the Italian V-FUCHS version to clinical changes. The significant postoperative improvements in questionnaire scores observed across both groups support the instrument’s sensitivity to changes in vision-related quality of life following surgical intervention [31,32,33]. These findings reinforce the utility of V-FUCHS not only as a diagnostic and monitoring tool but also as a valuable measure for capturing treatment outcomes in routine practice and research. Interestingly, no statistically significant difference in glare factor scores was observed between patients who underwent unilateral versus bilateral DMEK. This finding may be explained by the substantial improvement in glare symptoms achievable with unilateral DMEK alone, which can significantly enhance binocular visual function and reduce overall glare sensitivity [34]. These results underscore the ability of even a single-eye intervention to meaningfully impact patient-perceived visual quality.

In evaluating correlations between questionnaire scores and clinical parameters, both the VA and glare factors were associated with BCVA and CCT; however, only the correlation between the VA factor and BCVA reached statistical significance (*p* < 0.05). These findings support the concept that in FECD, corneal structural changes precede clinically detectable edema on CCT and contribute to increased light scatter, arising from guttae and subclinical corneal edema, which manifests as disability glare. As demonstrated with the original English-language V-FUCHS and its validated translations, glare-related symptoms can significantly impair visual function even when VA remains relatively preserved. These findings were further reinforced in our Italian cohort, highlighting the complementary and independent role of PROMs in clinical decision-making, as they provide clinically relevant information beyond traditional objective parameters. The Italian V-FUCHS provides a structured and patient-centered evaluation of visual symptoms, which can help identify individuals whose quality of life is significantly affected despite relatively preserved clinical findings. Together, these results underscore the potential utility of V-FUCHS in optimizing surgical timing and supporting more personalized treatment decisions, especially when traditional clinical metrics such as BCVA and CCT may not yet reflect the patient’s functional burden. Its integration into routine clinical workflows, as a pre-visit screening tool, could enhance its value in guiding ophthalmologist–patient discussions.

The additional strengths of the V-FUCHS instrument include its speed, simplicity, non-invasiveness, and cost-effectiveness, making it particularly well suited for routine clinical use. However, our study has some limitations, including a modest sample size and a lack of correlations with more clinical parameters, such as contrast sensitivity, wavefront aberration, and straylight measurement. Moreover, the instrument’s reduced sensitivity among patients with minimal or absent symptoms may limit its standalone role in very early FECD detection. This may reflect a potential floor effect, where individuals with a low symptom burden tend to score at the lower end of the scale, reducing the scale’s ability to detect subtle variations in symptom severity in this subgroup. Future studies should aim to validate these findings in larger and more diverse populations and further explore its associations with additional clinical metrics to enhance the interpretability and applicability of the V-FUCHS instrument. In particular, integrating objective optical parameters such as contrast sensitivity and wavefront aberration may help clarify the added value of the questionnaire in assessing optical damage to the cornea.

## 5. Conclusions

In conclusion, the Italian version of the V-FUCHS questionnaire is a valid and reliable PROM for assessing disease-specific visual disability in Italian-speaking patients with FECD. Our findings further support the clinical relevance of the V-FUCHS questionnaire in bridging the gap between patient-reported visual impairment and objective clinical measures. Subjective visual symptoms can have a highly individualized impact on vision-related quality of life, which may not be fully captured by standard ophthalmologic assessments. The V-FUCHS instrument provides valuable insight into the subjective impact of visual disability on daily activities, which is crucial in guiding the optimal timing for endothelial keratoplasty. As a quick, cost-effective, and non-invasive tool, its integration into routine clinical practice may aid in tailoring clinical and surgical decisions to the patient and evaluating their postoperative symptomatic improvement.

## Figures and Tables

**Table 1:** Demographic and clinical characteristics of included patients. BCVA = best-corrected visual acuity; CCT = central corneal thickness; DMEK = Descemet membrane endothelial keratoplasty; FECD = Fuchs’ endothelial corneal dystrophy.

		FECD		DMEK	
	Controls	Mild-to-Moderate	Advanced	Unilateral	Bilateral
Modified Krachmer grade	0	1–4	5–6	-	-
Participants (*n*)	20	18	15	9	12
Age (mean ± SD, years)	66.7 ± 10.8	63.6 ± 8.9	66.4 ± 10.2	69.7 ± 7.5	69.4 ± 7.5
Men (%)–Women (%)	10 (50%):10 (50%)	4 (22%):14 (78%)	11 (73%):4 (27%)	6 (67%):3 (33%)	5 (42%):7 (58%)
BCVA (mean ± SD, logMAR)	0.029 ± 0.050	0.056 ± 0.129	0.086 ± 0.112	0.043 ± 0.045	0.024 ± 0.038
CCT (mean ± SD, µm)	545 ± 14	550 ± 19	582 ± 35	548 ± 18	533 ± 23

**Table 2:** Item difficulty calibration and goodness-of-fit statistics. Items in the table are grouped according to their corresponding dimension to facilitate interpretation and analysis. The Italian translation has been presented next to the original English version (in square brackets) for better comprehension. Unipolar ordinal scales with 5 response categories were used to assess difficulty and frequency: * Quanto spesso hai ciascuna delle seguenti difficoltà? [How often do you have each of the following difficulties?] ** A causa della tua vista, quanta difficoltà hai [Because of your eyesight, how much difficulty do you have?]. In order to facilitate understanding, this clarification, consistent with the original version, has been added: “Se indossi occhiali o lenti a contatto per un’attività specifica, rispondi a tutte le seguenti domande come se stessi indossando i tuoi migliori occhiali o lenti a contatto, se necessario” [If you wear glasses or contact lenses for a particular activity, please answer all of the following questions as though you were wearing your best glasses or contact lenses if necessary].

			Infit	Outfit
	Mean	S.E.	Mean Squares	Mean Squares
**Visual Acuity Factor**				
Q6. * Nel complesso, diventa sempre più difficile vedere i dettagli più fini, ad esempio le foglie sugli alberi. [Overall, fine details are becoming harder to see, for example, leaves on trees.]	2.57	0.222	1.141	1.373
Q7. * Nell’ultimo mese, la mia vista ha interferito con le mie attività quotidiane. [During the past month, my vision interfered with my daily activities.]	2.48	0.191	1.244	1.124
Q8. ** Leggere un comune testo stampato su carta? [Reading ordinary print on paper?]	2.48	0.218	0.991	0.805
Q9. ** Leggere un testo su uno schermo? [Reading text on a screen?]	1.75	0.242	0.996	0.814
Q10. ** Svolgere lavori o hobby che richiedono una buona visione da vicino? [Doing work or hobbies that require you to see well up close?]	2.07	0.207	0.659	0.841
Q11. ** Leggere il testo sui contenitori dei medicinali e foglietti illustrativi? [Reading text on medicine bottles and package inserts?]	2.37	0.188	1.051	0.953
Q12. ** Vedere i prezzi dei prodotti quando fai spese? [Seeing the prices of items when shopping?]	1.75	0.242	0.855	0.688
**Glare factor**				
Q1. * Nell’ultimo mese, ho notato dei cambiamenti della mia vista nel corso della giornata. [During the past month, my eyesight changed over the course of the day.]	1.88	0.199	0.811	0.614
Q2. * Nell’ultimo mese, ho avuto una visione annebbiata, peggiore al mattino. [During the past month, I have had blurred vision that is worst in the morning.]	2.22	0.209	0.798	0.793
Q3. * Nell’ultimo mese ho avuto difficoltà a mettere a fuoco, peggiore al mattino. [During the past month, I have had trouble with focusing that is worst in the morning.]	1.92	0.201	0.851	0.698
Q4. * Di notte, le luci brillanti sembrano circondate di raggi luminosi. [At night, bright lights look like a starburst]	2.16	0.181	1.336	1.238
Q5. * Di notte, attorno alle luci appare un cerchio luminoso (alone), ad esempio attorno ai lampioni delle strade. [At night, a bright circle (halo) appears to surround lights, such as street lights.]	2.39	0.187	1.273	1.295
Q13. ** Vedere ciò che hai di fronte quando entri da una zona illuminata dal sole in un’area in ombra, come ad esempio entrando in un parcheggio sotterraneo? [Seeing what is ahead of you when you enter from daylight into a shady area, such as entering into a parking ramp?]	1.50	0.191	0.960	1.025
Q14. ** Vedere ciò che hai di fronte di notte quando un’auto in arrivo ha i fari accesi? [Seeing what is ahead of you when an oncoming car has headlights on at night?]	1.67	0.170	0.805	0.931
Q15. ** Vedere ciò che hai di fronte quando il sole è basso all’alba o al tramonto? [Seeing what is ahead of you when the sun is low during sunrise or sunset?]	2.22	0.182	1.262	1.158

## Data Availability

The raw data supporting the conclusions of this article will be made available by the authors on request.
